# Horizons in Veterinary Precision Oncology: Fundamentals of Cancer Genomics and Applications of Liquid Biopsy for the Detection, Characterization, and Management of Cancer in Dogs

**DOI:** 10.3389/fvets.2021.664718

**Published:** 2021-03-23

**Authors:** Jason Chibuk, Andi Flory, Kristina M. Kruglyak, Nicole Leibman, Alexis Nahama, Nilesh Dharajiya, Dirk van den Boom, Taylor J. Jensen, Jeffrey S. Friedman, M. Richard Shen, Francisco Clemente-Vicario, Ilya Chorny, John A. Tynan, Katherine M. Lytle, Lauren E. Holtvoigt, Muhammed Murtaza, Luis A. Diaz, Dana W. Y. Tsui, Daniel S. Grosu

**Affiliations:** ^1^PetDx, La Jolla, CA, United States; ^2^The Cancer Institute, Animal Medical Center, New York, NY, United States; ^3^ARK Animal Health, San Diego, CA, United States; ^4^Healthbit.ai Inc., San Diego, CA, United States; ^5^Advisor to PetDx, San Diego, CA, United States; ^6^Laboratory Corporation of America, Durham, NC, United States; ^7^Friedman Bioventure, Inc., San Diego, CA, United States; ^8^RS Technology Ventures LLC., Rancho Santa Fe, CA, United States; ^9^La Merced Veterinary Oncology, Calpe, Spain; ^10^Department of Surgery and Center for Human Genomics and Precision Medicine, University of Wisconsin-Madison, Madison, WI, United States; ^11^Division of Solid Tumor Oncology, Memorial Sloan Kettering Cancer Center, New York, NY, United States

**Keywords:** dog, cfDNA, cell-free DNA, circulating tumor DNA, cancer, genomic, liquid biopsy, one health

## Abstract

Cancer is the leading cause of death in dogs, in part because many cases are identified at an advanced stage when clinical signs have developed, and prognosis is poor. Increased understanding of cancer as a disease of the genome has led to the introduction of liquid biopsy testing, allowing for detection of genomic alterations in cell-free DNA fragments in blood to facilitate earlier detection, characterization, and management of cancer through non-invasive means. Recent discoveries in the areas of genomics and oncology have provided a deeper understanding of the molecular origins and evolution of cancer, and of the “one health” similarities between humans and dogs that underlie the field of comparative oncology. These discoveries, combined with technological advances in DNA profiling, are shifting the paradigm for cancer diagnosis toward earlier detection with the goal of improving outcomes. Liquid biopsy testing has already revolutionized the way cancer is managed in human medicine – and it is poised to make a similar impact in veterinary medicine. Multiple clinical use cases for liquid biopsy are emerging, including screening, aid in diagnosis, targeted treatment selection, treatment response monitoring, minimal residual disease detection, and recurrence monitoring. This review article highlights key scientific advances in genomics and their relevance for veterinary oncology, with the goal of providing a foundational introduction to this important topic for veterinarians. As these technologies migrate from human medicine into veterinary medicine, improved awareness and understanding will facilitate their rapid adoption, for the benefit of veterinary patients.

## Introduction

Cancer is frequent in dogs and is by far their most common cause of death (1–5). While dogs and humans have a similar lifetime risk of cancer (between 1:2 and 1:4), dogs have an annual incidence of cancer that is up to 10-fold higher than in humans, as their lifetime risk is compressed into a much-abbreviated lifespan (1, 2). Similar to humans, both genomic and environmental factors drive cancer incidence in dogs: cancer predisposition mutations are concentrated in many breeds as an inadvertent side effect of selective breeding; and dogs share the same environment as humans, including exposure to many carcinogens (6, 7). These considerations help explain why ~4–6 million dogs are newly diagnosed with cancer per year in the US in a population of under 90 million as compared to 1.8 million cancer diagnoses in humans in a population of ~330 million (8). Like humans, the burden of cancer in dogs increases with age: up to 50% of dogs over 10 years of age will develop cancer during the remainder of their lives (3, 9, 10).

Canine cancer also carries a significant mortality risk (3, 8, 11), since many canine cancers are diagnosed at advanced stages after there has been microscopic (12, 13) or macroscopic spread (12, 14–16) and a cure is no longer achievable. With rising pet ownership and increased emotional attachment to pets, the substantial burden of canine cancer goes well-beyond the immediate health implications for the dog, with significant emotional and financial impact on dog owners (17–21). Given the high incidence of cancer in dogs, all companion animal practices are exposed to oncology cases on a regular basis, and cancer care is an essential part of pet health care (13).

Over the past decade, genomic medicine has made great strides thanks to technological breakthroughs such as the introduction of next generation sequencing (NGS). In 2005, the National Institutes of Health (NIH) launched The Cancer Genome Atlas (TCGA), a landmark initiative aiming to molecularly characterize the genomic landscape of human cancer (22). By 2013, TCGA concluded enrollment with over 20,000 samples and built a knowledge base across all major human cancer types (22, 23). This effort, together with similar international initiatives such as the International Cancer Genome Consortium (24), enabled rapid cancer biology research and helped facilitate the development of new molecularly targeted therapeutic agents for cancer. As a result, tumor tissue-based molecular testing has become an integral part of the “precision medicine” trend in cancer care for humans (25). More recent innovations in the field have enabled non-invasive testing based on a simple blood draws; typically referred to as “liquid biopsy,” this type of testing most commonly relies on analysis of cell-free DNA (cfDNA) fragments released by the tumor cells into the bloodstream and known as circulating tumor DNA (ctDNA) (26–30).

**Figure 1:**
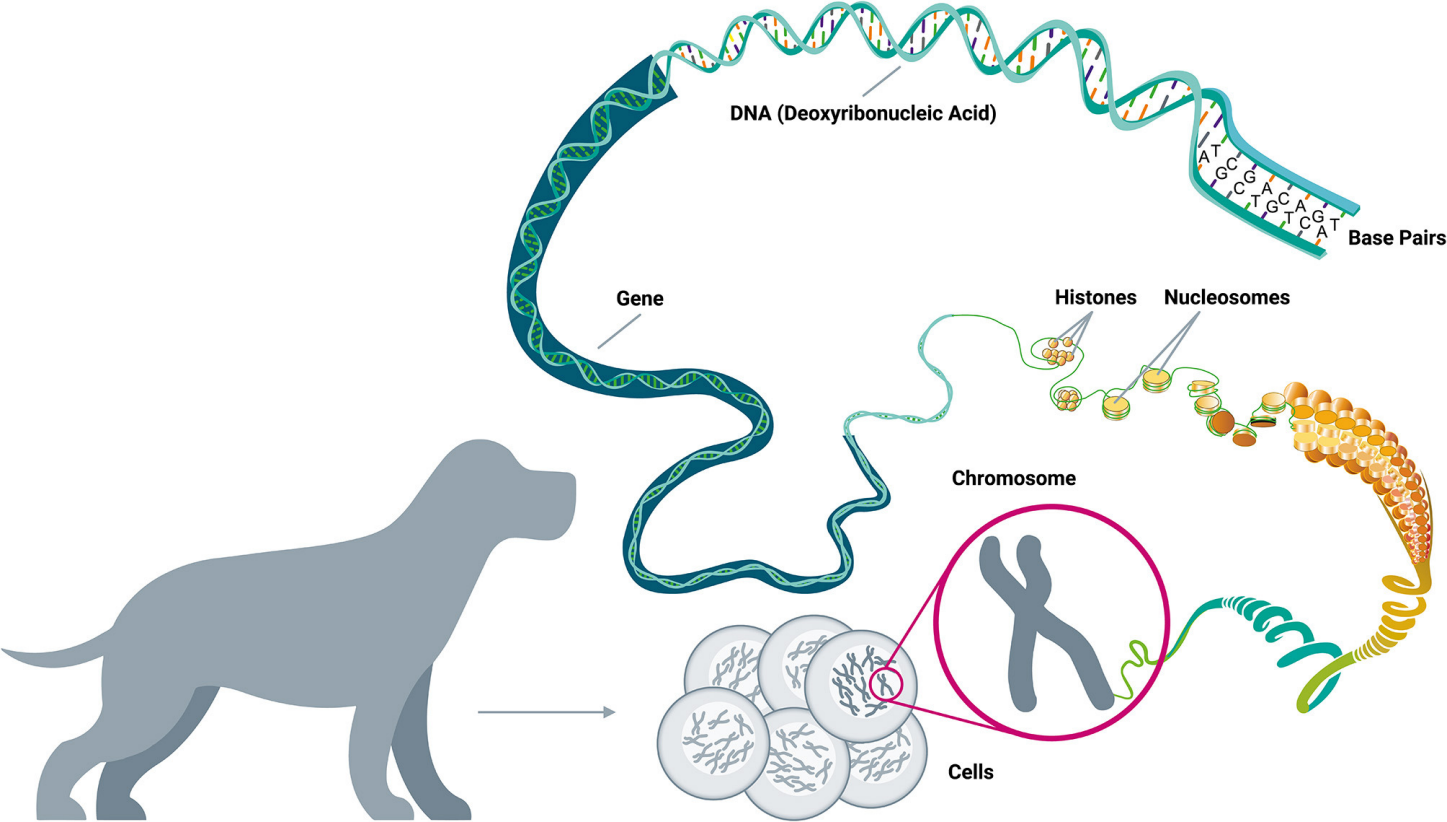
*A brief guide to genomics*. Cancer is a disease of the genome because DNA alterations provide the biological basis of cancer. Each body cell (except for mature red blood cells) contains a full copy of the organism's genome within a set of chromosomes packed in its nucleus. The DNA double-helix is formed by four nucleotides, or bases, assembled in complementary pairs via hydrogen bonds: adenine (A) is always paired with thymine (T), and cytosine (C) is always paired with guanine (G). The gene is the basic unit of heredity and consists of a long sequence of nucleotides that encodes for the synthesis of a protein by transcription to RNA (ribonucleic acid) in the cell's nucleus, followed by translation to a sequence of amino acids in the cytoplasm. The average gene comprises several thousand bases, with wide size variation. The DNA double-helix strand wraps around a set of histone proteins, forming structures known as “nucleosomes” at regular intervals along the length of the strand (Adapted from National Human Genome Research Institute, genome.gov).

The first canine reference genome was published in 2005 (31), not long after the publication of the human reference genome (Figure 1) (32–34). However, progress in canine genomics has not been nearly as rapid as in humans, and most advances in genomic medicine have not yet been adopted in veterinary medicine. Certain areas of canine genetics have seen meaningful progress, including breed identification (35, 36), breed-specific disease predisposition (37–39), and genetic determinants of heritable disorders (40). Much of this accumulated knowledge is now available to veterinarians and pet owners through commercial testing options. However, only a small fraction of the scientific progress made in humans has been transferred into the arena of canine oncology. More research and development pertaining to the genetic predispositions underlying canine cancer syndromes, and to the detection, characterization, and management of cancer in dogs, is urgently needed to allow the standard of cancer care in veterinary medicine to catch up with human medicine standards.

With a few notable exceptions—such as *BRAF* testing in urine for detection of canine urinary tract cancer (41), and testing for *c-kit* mutations in mast cell tumors (42–44)—the field of veterinary oncology has yet to utilize the full power of genomics for its precision medicine benefits. However, the rapid adoption of genomics-based testing by the veterinary community could pose risks due to the current lack of regulatory oversight for high complexity molecular testing. Clinical genomic testing for veterinary applications can be currently marketed without any peer-reviewed clinical validation studies, or based on studies in small cohorts that may not be representative of the intended-use population (45). There is currently no established regulatory approval pathway in the United States for veterinary diagnostics, and while a form of accreditation is available through the American Association of Veterinary Laboratory Diagnosticians, this accreditation is limited to publicly funded, full-service laboratories and is not available to privately owned commercial labs (46, 47). In the United States, laboratories conducting high complexity molecular testing in humans must secure certification under CLIA (Clinical Laboratory Improvement Amendments) and may also pursue accreditation through CAP (College of American Pathologists); many laboratory-developed tests (LDTs) intended for oncology applications are also regulated by the US FDA (Food and Drug Administration) (48–50). Because no such standards exist for high complexity molecular testing in veterinary medicine, low-quality tests could easily find their way into clinical use, leading to poor outcomes for patients. The lack of external oversight in veterinary diagnostic testing means that it is critically important for highly complex, novel tests to undergo rigorous analytical and clinical validation, with detailed findings published in peer-reviewed journals for full transparency (51).

To develop reliable genomics-based testing solutions for veterinary applications, significant research and development efforts will be required. This is especially true for blood-based liquid biopsy tests since the proportion of ctDNA in the plasma can be very low and variable, requiring highly sensitive detection with minimal false positive results (52). Analytical validation of any such test must evaluate the entire process – from blood collection to shipping, accessioning, separation of plasma and buffy coat (white blood cell - WBC) components, DNA extraction and sequencing library preparation, data generation by NGS, and sophisticated bioinformatics analysis – through adequately designed and powered studies (53). Clinically, the test will need to be validated for each intended use. The unique, non-invasive nature of liquid biopsy allows it to be deployed in multiple clinical use cases across the full spectrum of cancer care in dogs, including: (1) screening for early detection in patients without any signs of cancer; (2) aid in diagnosis in patients with suspected cancer; (3) molecular profiling for targeted treatment selection; (4) detection of minimal residual disease after curative-intent interventions; (5) treatment response monitoring; and (6) recurrence monitoring in patients who achieve complete remission after initial treatment. Each of these use cases will require independent clinical validation in the corresponding intended-use population, with clinical utility ultimately determined by the test's demonstrated ability to inform clinical decision-making or improve clinical outcomes in each use case.

This article will review fundamental principles of cancer genomics for a contemporary understanding of cancer as a disease of the genome; describe key biological and technical considerations for developing and validating a liquid biopsy assay for veterinary cancer applications; and conclude with a review of the six clinical use cases for liquid biopsy described above. Armed with a well-informed appreciation for the validation requirements and the potential of liquid biopsy solutions to significantly improve care for their patients, veterinarians will be well-positioned to evaluate and employ validated liquid biopsy tests as they enter the clinic in the coming years. Once developed and commercialized, liquid biopsy solutions promise to usher in a new era for veterinary medicine, enabling personalized cancer care for pets at the same level of quality and sophistication already available to humans at major cancer centers today.

## Fundamentals of Cancer Genomics

### Cancer as a “Disease of the Genome”

Historically, cancer has been defined by its organ or tissue of origin, or by its cellular characteristics, as the ability of clinicians to understand and describe it was limited to gross examination and/or microscopic evaluation. Advances in molecular medicine over the past two decades have revealed that normal cells accumulate random genomic alterations over time as a result of DNA replication errors, as well as exposure to endogenous factors (such as free radicals) and to environmental (exogenous) carcinogens such as various forms of radiation and mutagenic chemicals in food and air (54–57); and that cancer results when one or more of these alterations confer an uncontrolled growth advantage to a population of cells (58). These random alterations are called *somatic alterations*, as they are acquired “in the body” after birth; in some cases, cancer-predisposing alterations are already present at birth, having been inherited from parents as *germline alterations*.

Most somatic alterations are promptly corrected by intracellular DNA repair mechanisms or (if unrepaired) are severe enough to trigger death of the affected cell, with no ill consequences for the organism; however, when such alterations occur in specific locations in the genome, and are not corrected, a chain of events is set in motion that ultimately leads to the development of cancer. Such alterations confer a growth and/or survival advantage to the affected cells, either by triggering increased cell replication or by inhibiting the processes that keep cell division in check; these are analogous to pressing the gas pedal and cutting the brakes on a car, respectively. Tumor growth can be further accelerated by the accumulation of new somatic alterations with the passing of time; this causes cancer cells to replicate faster, invade surrounding tissues, travel to distant organs by lymphatic and vascular routes, and evade the immune system's surveillance and control mechanisms. When the number of cancer cells reaches around one billion, the malignant mass is ~1 cm in size and weighs about 1 g (59, 60); at this stage, the mass typically becomes detectable by physical and imaging examinations, and may have already started to cause clinical signs such as bleeding, lameness, weight loss, lethargy, etc. This clinical manifestation is called cancer, and is commonly described by its organ of origin, size, and appearance under the microscope (histological diagnosis and grading). The tumor spread is defined by the TNM (tumor, node, and metastasis) staging system. Fundamentally, however, cancer is a disease of the genome, as it is directly caused by genomic alterations and cannot develop in the absence of such alterations (61).

### Genomic Alterations in Cancer

As malignant tumors grow, they develop the ability to invade adjacent areas and metastasize to distant locations in the body through the accumulation of DNA alterations in key genes (58). A primary “gatekeeping” alteration provides an initial growth advantage and allows the affected cell to replicate more quickly than the surrounding cells, becoming a microscopic clone (58); in time, a cell within this clone will randomly acquire a second alteration, typically in another gene, and initiate a subsequent round of clonal expansion with enhanced selective growth advantage for the cells containing both alterations. In this way, the process of novel mutation acquisition followed by clonal expansion continues, leading to the evolution of malignant subclones that can invade surrounding tissues, metastasize to lymph nodes, and spread to distant organs (58).

Genomic alterations that confer a selective growth advantage are termed *driver mutations*. The cumulative effect of this advantage, over many cell divisions, results in a mass of billions of malignant cells growing at an accelerating rate, with multiple subclonal populations emerging through the successive accumulation of additional mutations. In humans, this is a process that begins with a single driver mutation and ends with metastatic disease, and is estimated to take decades (58). On average, a human cancer genome contains 4–5 driver mutations, though there is wide variability across different cancer types (62). Cancer genomes also contain somatic alterations that do not confer a discernible growth advantage to the cell and are referred to as *passenger mutations* (58). Detection of either class (*driver* or *passenger*) can point to the presence of cancer, but only driver mutations can inform the selection of effective targeted therapies (58, 63).

**Figure 2:**
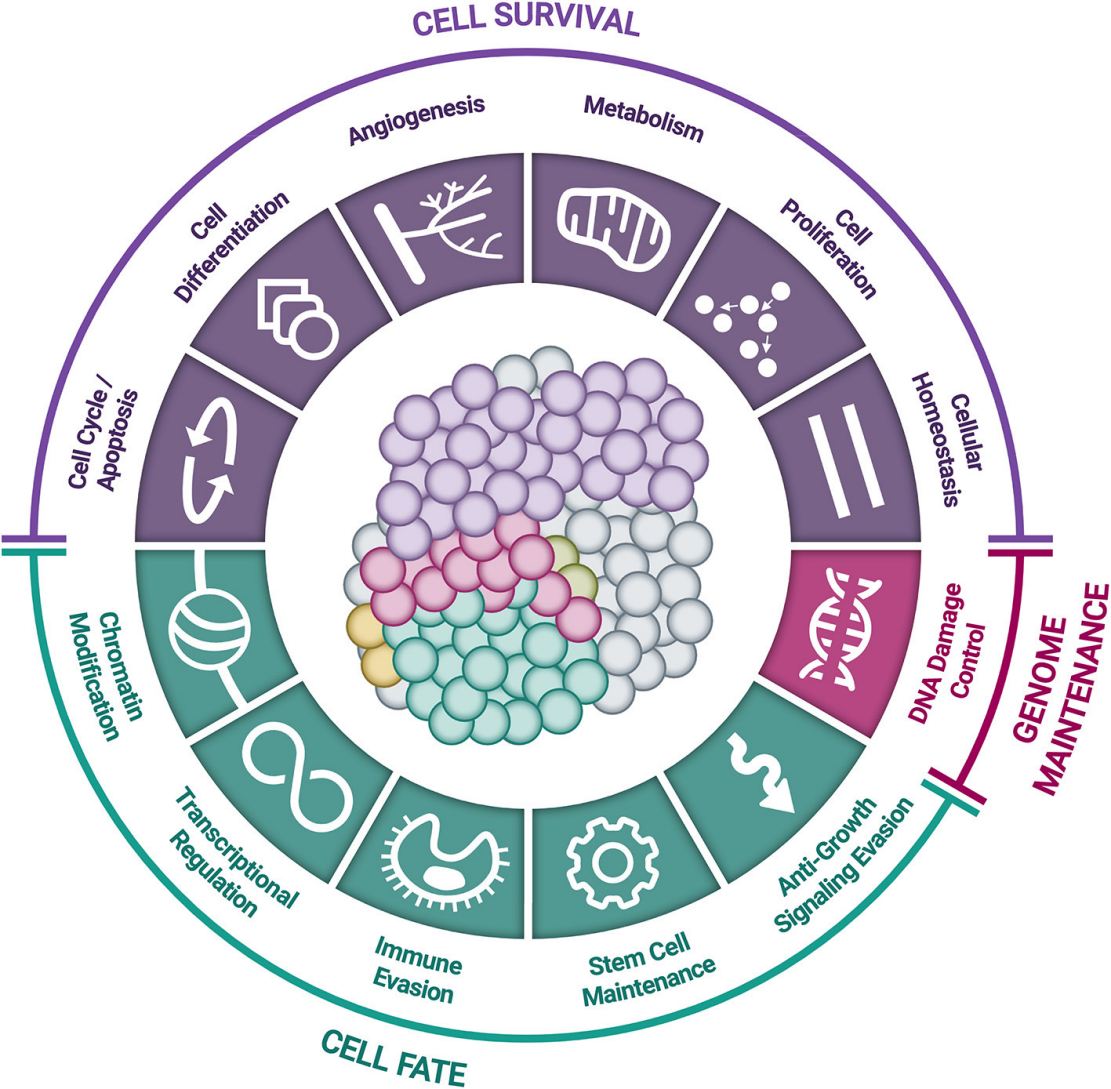
*Cellular pathways and functional processes involved in cancer*. Driver mutations in cancer-related genes are responsible for cancer development. These cancer-related genes are implicated in 12 cellular signaling pathways, which can be grouped into 3 core cellular functions: cell survival, cell fate, and genome maintenance [Inspired by Hanahan & Weinberg (2011) and Vogelstein et al. (2013)] (58, 68).

Driver mutations are not randomly distributed across the genome; in fact, of the more than 20,000 human genes, fewer than 1,500 have been implicated in cancer development (58, 64–67). These cancer-related genes are implicated in 12 specific cellular pathways (Figure 2), which in turn relate to three main functions: (1) *cell survival* (ability to thrive in nutrient-poor conditions, dysregulation of apoptosis, angiogenic stimulation); (2) *cell fate* (division and differentiation); and (3) *genome maintenance* (ability to survive despite gross chromosomal abnormalities, acceleration of mutation acquisition, and DNA damage control) (58, 68, 69).

As noted previously, cancer-related alterations can be either somatic (acquired after birth, and present in only a subset of cells in the body) or germline (inherited, and present in every cell). Germline alterations resulting in cancer predisposition - for example *BRCA1* and *BRCA2* variants - increase the risk of breast cancer in humans, and alterations in these genes have also been documented in dogs with mammary tumors (70). In humans with cancer-predisposing germline alterations, the diagnosis is often made at a younger age than is typical for that cancer type, and therefore these patients benefit from proactive cancer screening that can detect such cancers at earlier stages (71). As researchers learn more about heritable canine cancer risk, proactive cancer screening in younger dogs, informed by the presence of germline alterations, will likely demonstrate increasing clinical utility and lead to better clinical outcomes.

**Figure 3:**
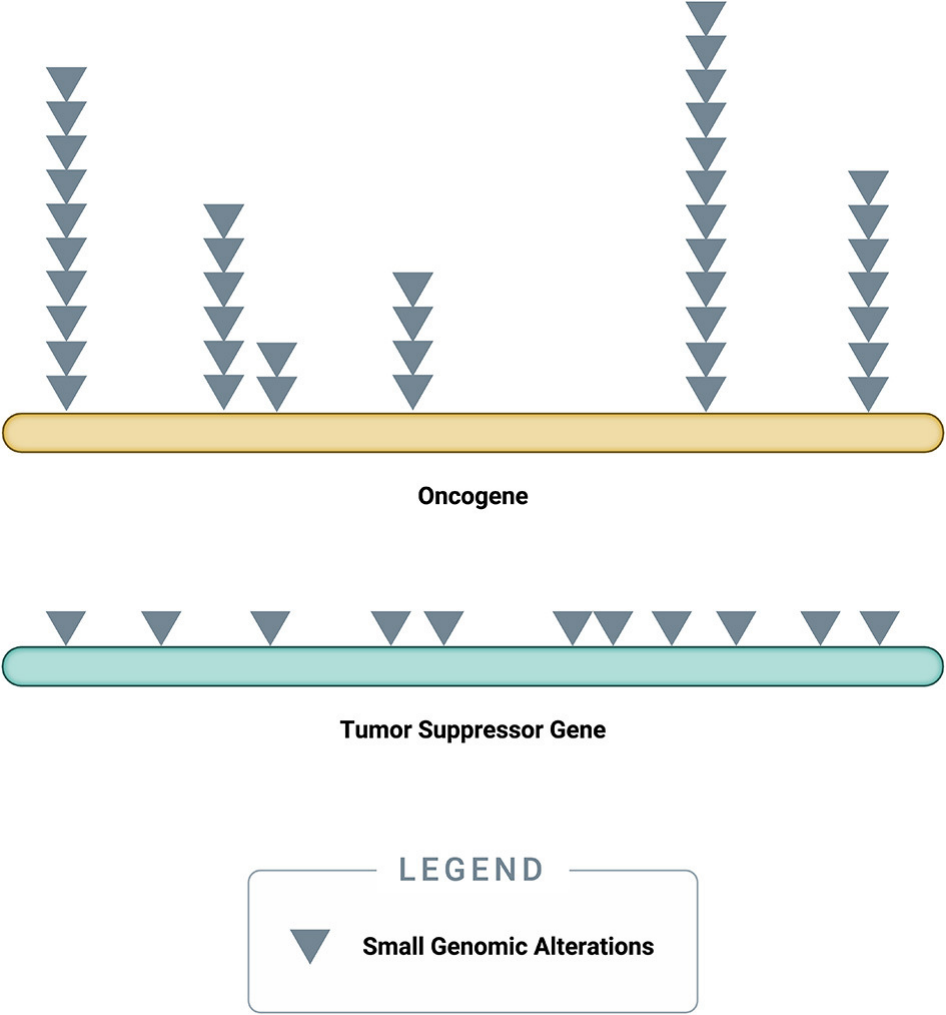
*Accumulation of small genomic alterations in cancer-related genes*. Small genomic alterations in oncogenes tend to be activating mutations, which cluster at very specific locations (“hotspots”), whereas small genomic alterations in tumor suppressor genes (TSGs) tend to be inactivating mutations and may occur across the full length of the gene. The design of a high-quality genomic assay needs to account for these characteristics in order to identify relevant alterations across cancer-related genes in an efficient manner.

Somatic driver mutations predominantly occur in two types of genes: oncogenes and tumor-suppressor genes (TSGs) (Figure 3) (58). Oncogenes typically acquire *activating* (or gain of function) mutations in very specific locations (known as “hotspots”); these activating mutations increase the rate of cell division, inhibit programmed cell death (apoptosis), or help the cell evade immune surveillance (58). TSGs, on the other hand, typically acquire *inactivating* (or loss of function) mutations, which can occur across the full length of the gene (58). As their name implies, TSGs serve as a built-in control mechanism to suppress the development and growth of tumors; inactivating mutations impair this critical protective function, leaving oncogene-driven cancers to grow unchecked (58).

Successive genomic alterations can accumulate in both oncogenes and TSGs, thereby accelerating the progression of the disease in advanced stages of cancer (72). Early in cancer formation, however, disease progression occurs at a relatively slow pace (72). In humans, many tumors grow over 10 to 30 years before clinical manifestation and remain confined to the organ of origin through most of this period (72). This timeframe represents a considerable window of opportunity for early detection that can allow for a cure to be achieved by simple surgical removal of the localized mass (72–74). This paradigm holds true in canine cancer as well: in some types of canine cancers, for example mast cell tumors and soft tissue sarcomas, clinical outcomes are often excellent with early detection and proper surgical excision (75, 76).

Each patient's cancer is characterized by a variety of genomic alterations, and even within a particular cancer type (breast, colon, etc.), no two cancers are the same (77). There is no established 1:1 correspondence between a given tumor type and a given genomic alteration. For example, the *BRAF* V600E mutation is most commonly seen in human melanoma but is also seen in other cancers (78); likewise, its canine ortholog V595E is common in transitional cell carcinoma but is also present in different canine cancer types (79). The presence of the same mutation in different cancer types may have different therapeutic implications. For example, in humans, targeting *BRAF* with the agent vemurafenib works more effectively in melanoma than in other cancer types (80). Significant amounts of focused research will be required to understand the efficacy of various targeted agents in specific canine cancers.

Cancer in adult humans typically has dozens to hundreds of mutations per case, while pediatric cancers usually have far fewer mutations per case (58). A commonly employed metric for describing the frequency of mutations in a given cancer case is the tumor mutational burden (TMB), represented by the number of mutations per Mb (megabase, i.e., one million DNA bases) (81). A recent review of over 100,000 human cancer cases across more than 500 cancer types revealed a wide TMB spectrum, ranging from 0 to over 1,000 mutations/Mb, with a median of 3.6 mutations/Mb and increasing with patient age (82). Although less extensively studied, canine cancer genomes have been shown to exhibit similar TMBs in published studies, with a median of 1.98 mutations/Mb in canine osteosarcoma (83), 2.04 mutations/Mb in primary canine lung cancer (84), and a range of 0.1–2.1 mutations/Mb in canine hemangiosarcoma (85, 86). TMB has been shown to be a marker for predicting response to immunotherapy in humans, with high-TMB tumors more likely to respond (87, 88). The ability to non-invasively measure TMB from a blood sample could gain clinical relevance as immunotherapies become increasingly utilized in the management of canine cancers (10).

The extreme diversity of genomic features across cancer types, coupled with the fundamental understanding of cancer as a “disease of the genome,” have opened the door to novel diagnostic approaches that go beyond the notion of a specific test for a specific type of cancer and favor a “pan-cancer” model where a single, highly complex diagnostic assay can be used to detect and characterize a broad range of cancer types (27, 28, 61).

### Classes of Genomic Alterations

**Figure 4:**
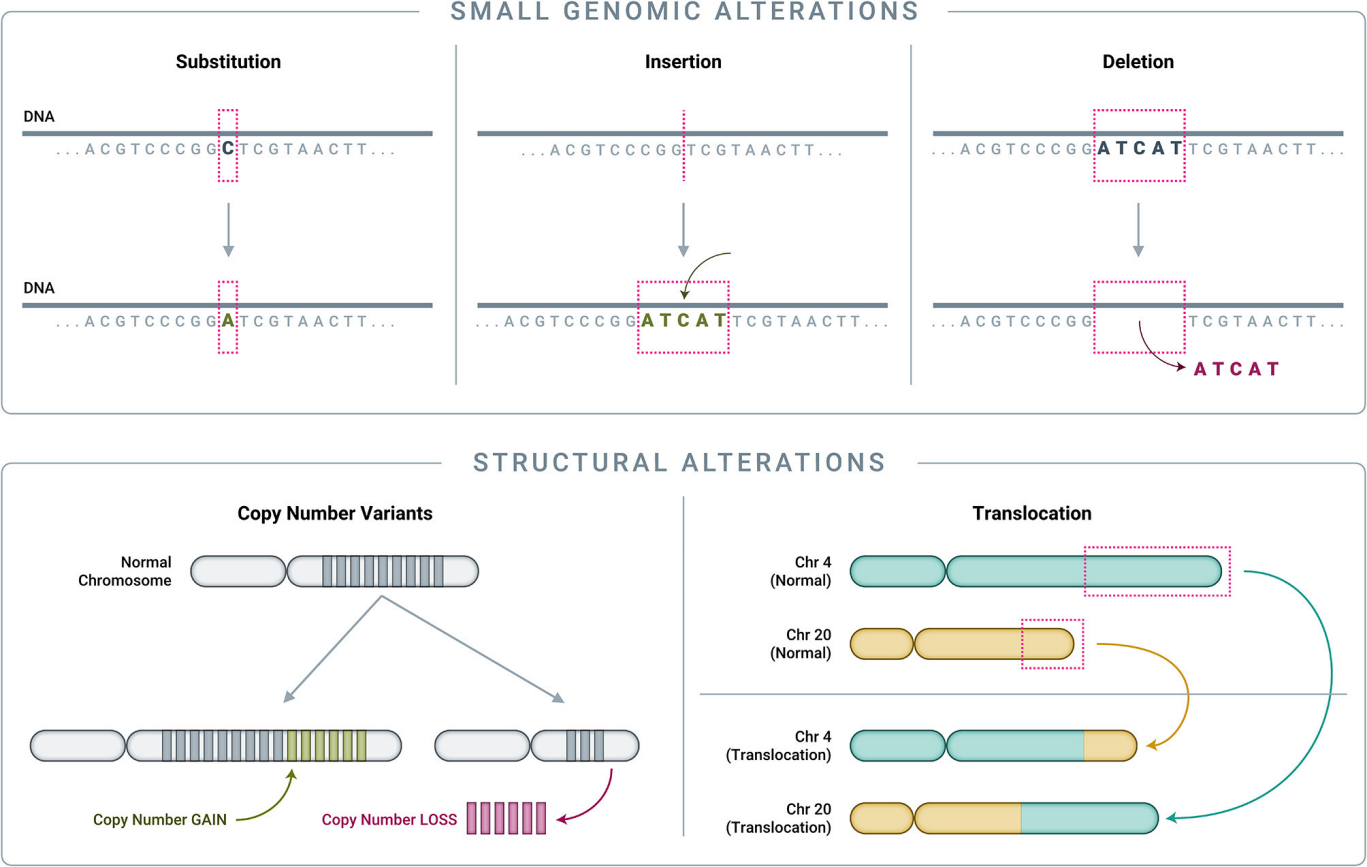
*Classes of genomic alterations*. Small genomic alterations include single nucleotide variants (SNVs) as well as small insertions and deletions (collectively known as “indels”). SNVs arise when one nucleotide is substituted for another, which can result in altered amino acid translation and an altered protein product. Indels involve the insertion or deletion of one or more nucleotides from the normal DNA sequence, resulting in an altered protein product. On a much larger scale, structural alterations typically involve thousands to millions of nucleotides. Copy number variants (CNVs) are a common type of structural alteration, involving gains or losses of large stretches of DNA. Translocations represent another type of structural alteration, whereby two distant, otherwise unrelated genomic regions are joined together, creating “gene fusions” that can drive tumor growth.

To understand how genomics-based testing can characterize cancer accurately, it is important to first review the main classes of genomic alterations that drive cancer initiation and progression (Figure 4). Though counter-intuitive, many cancers are driven by single nucleotide variant (SNV) “hotspot” alterations that involve a change of just one letter out of several billion letters in the genome (58, 89–91). Another class of small genomic alterations are indels (insertions and deletions), in which one to several nucleotides are inserted into, or removed from, the normal DNA sequence (89–91). Larger genomic events, affecting thousands to millions of nucleotides and known as structural alterations, can also cause significant genomic disruption, leading to cancer (92). Cancer-related structural alterations include: (1) copy number variants (CNVs), in which large segments of DNA (thousands to millions of bases long, up to entire chromosomes) are either completely absent or are abnormally repeated, and (2) translocations, in which DNA strands from unrelated parts of the genome are joined together and result in “fusion genes” in the RNA transcript (92).

Numerous studies have revealed that the disease etiology of a given cancer is typically driven either by focal somatic alterations (SNVs, indels, and/or translocations) or by CNVs, but rarely by both categories (58, 93, 94). This association with specific classes of driver genomic alterations is often cancer type or subtype-specific, with cancers such as sarcomas—which are far more common in dogs than in humans (9)–being mostly CNV-driven while others, such as carcinomas of the lung or gastrointestinal tract, being mostly SNV and indel-driven (93).

### Clonality and Tumor Evolution

**Figure 5:**
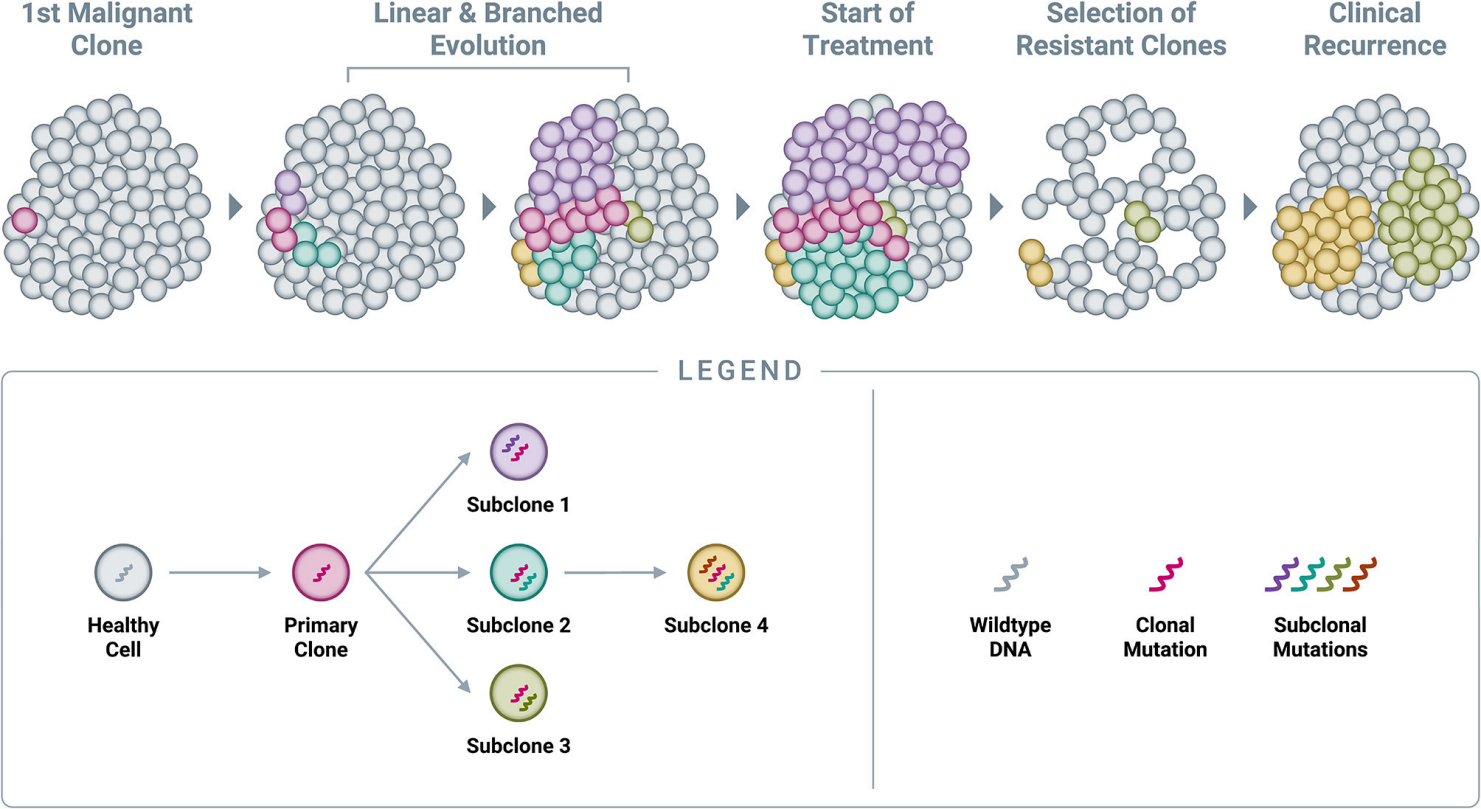
*Accumulation of genomic alterations and emergence of resistance*. Cancer begins with a single genomic alteration in a cancer-related gene, which provides a selective growth advantage that allows the cell with the original “clonal mutation” (also known as the “truncal mutation”) to grow and divide more quickly than neighboring healthy cells. Over time, additional genomic alterations accumulate in the DNA of these cancerous cells, leading to both linear and branched evolution from the original clonal population. This leads to a tumor comprised of various subclones, all of which share the original truncal mutation but also feature additional, unique mutations (known as “private mutations”). Administration of an efficacious treatment will typically eliminate many cells in the tumor, resulting in a reduction in tumor burden and clinical remission; however, certain subclones already harboring resistance mutations will often survive treatment at clinically undetectable levels and subsequently expand in the absence of competition. In time, this leads to the clinical observation of recurrence.

By the time cancers are diagnosed, they are typically large—measuring centimeters in diameter—and thus comprised of billions of cells (59, 60). As described previously, cancer growth is characterized by the successive accumulation of somatic alterations, meaning that tumors are not static—they constantly evolve to include additional alterations beyond the original clonal (or “truncal”) alteration (Figure 5) (58). At the time of diagnosis, when the primary tumor is one or more centimeters in size, most patients do not in fact have “cancer”; rather, they have “cancers,” as the disease has already evolved to consist of multiple sub-populations of cells (subclones), each sharing the original clonal alteration but further evolved with its own additional unique mutational profile. This phenomenon is known as spatial heterogeneity, which can manifest as intratumor heterogeneity (within a single primary or metastatic tumor mass) and/or intrapatient heterogeneity (between different tumor masses within the same individual) (58, 95–98). Once seeded in a new location, metastatic deposits subsequently accumulate additional alterations, which can be distinct from those present in the primary tumor (58). New alterations, which are unique to a specific subclone within the primary tumor or at a metastatic site, are referred to as *private mutations* (95).

At the time of diagnosis under the current standard of care, a single biopsy of a single tumor will only reveal a set of mutations at one point in time for that one specific physical location in the tumor. However, it is likely that an adjacent area in the primary tumor, or a distant metastatic site, will have a different set of mutations (95, 99). As cancer therapeutics become increasingly guided by the tumor's molecular alterations, a representative and unbiased view of the mutational landscape across all subclones in the body will be essential (100).

Treatment success is currently determined by observing a reduction or apparent disappearance of the tumor mass on imaging or physical examination, but in many cases this is ultimately followed by reemergence of the cancer at the same anatomic site or elsewhere. From a molecular perspective, the treatment may have been successful in eliminating a large subset (perhaps the dominant clone) of cancer cells with a particular genomic signature but left behind other subclones that harbored private resistance mutations to the treatment (Figure 5) (101). The treatment-resistant cell populations (subclones) were likely already present in the tumor at the time of initial treatment, albeit in smaller numbers compared to the dominant clone; once the overall disease burden is reduced as a result of treatment pressure on the susceptible clone, these resistant subclones are allowed to prosper, with reduced competition for space and nutrients from the previously dominant clone (58, 102). This highlights an important benefit of detecting cancer earlier, before it accumulates a more diverse clonal composition that may increase its overall resistance to treatments.

In humans, this accumulation of additional somatic alterations is known to progress at relatively predictable rates. By the time a tumor reaches a clinically detectable size (typically 1 g, or 1 cm^3^, or 1 billion cells), it has undergone 30 volume doublings (103); the time that the tumor has been present in the body can be roughly estimated by back calculation via the tumor doubling time (TDT), if known. In human breast tumors across multiple subtypes, median tumor volume doubling times of 85–185 days have been reported (104). Assuming constant growth rates, the average breast cancer would need many years to reach a size at which it could be clinically detected. Currently recommended screening intervals in humans take these tumor growth estimates into account. For example, screening for breast cancer with mammography is recommended every 1–2 years beginning at age 45–50 (105, 106), while screening for colorectal cancer is recommended every 3–5 years beginning at age 50 (107). In effect, these recommendations reflect current understanding of the growth rates of specific cancers from early stage to late stage in humans. Routine screening at set intervals also provides the benefit of “cumulative detection”—the combination of detection rates compounded over time, such that after 2+ cycles of screening, the overall detection rate will be higher than if a single screening test were used at just one point in time (108, 109).

The rates of growth of various cancer types in dogs are not as well understood as in humans; however, given the shorter canine lifespan, it can be assumed that the time from a cancer's molecular inception to clinical manifestation is significantly compressed. Though TDTs have been rarely reported in veterinary medicine, those that have been reported support this assessment: for example, the mean TDT for induced canine lung adenocarcinomas was ~100 days, and for human pulmonary adenocarcinoma was greater than 1 year (110, 111). As in humans, TDT is important for informing the cadence of cancer screening in dogs; given these preliminary estimates, an annual or semiannual screening interval, when such testing becomes available, should allow for the detection of a significant proportion of canine cancers at the localized (resectable) stage.

### Comparative Oncology: Dogs and Humans

Comparative oncology is typically described as the study of naturally occurring cancers in veterinary patients to benefit both humans and animals, through the study of cancer biology, pathogenesis, and treatment (112). Canine and human cancers share many histological, molecular, physiological, and even epidemiological features, and this commonality provides the rationale for the field of comparative oncology, wherein a deeper understanding of cancer in one species can drive corresponding insights in the other (7, 113–115). Dogs represent a powerful model system for the study of human cancers and vice versa, as cancers occur spontaneously in both species and are driven by orthologous genomic changes that impact corresponding biological pathways (114, 116, 117).

The human genome is ~3.1 billion nucleotides in length; the canine genome is ~20% smaller at ~2.4 billion nucleotides (31–33, 66, 118). Despite the size difference, the human and canine genomes have a high degree of homology (estimated at around 85%) (31); and among the top 100 human genes most frequently mutated in cancer, the extent of homology in the canine genome is likely even higher. Despite these commonalities, there are important differences between human and canine cancers, and these differences can be intelligently leveraged to drive faster translation of discoveries from one species to the other. For example, while dogs and humans are susceptible to cancers throughout the body, some cancers that are common in dogs are rare in humans (e.g., osteosarcoma, T-cell lymphoma) (83, 119, 120), and vice versa (Table 1). It is difficult to perform well-powered studies in rare cancer types; however, research efforts can progress faster in the species where the cancer is more common, and key insights can be translated back to the other species.

**Table 1:** Common cancers in humans and dogs (8, 13).

**Common cancers in humans**	**Common cancers in dogs**
• Bladder cancer • Breast cancer • Colorectal cancer • Kidney cancer • Lung cancer • Skin cancer^*^ • Non-Hodgkin Lymphoma • Prostate cancer	• Anal sac carcinoma • Lymphoma • Mammary gland cancer • Mast cell tumor • Oral malignant melanoma • Osteosarcoma • Soft tissue sarcoma • Splenic hemangiosarcoma

* *Including melanomas as well as basal cell and squamous cell skin cancers*.

## Liquid Biopsy: The Next Frontier in Veterinary Cancer Care

Liquid biopsy broadly refers to the sampling and analysis of analytes from various biological fluids (primarily blood, but in some cases also urine, cerebrospinal fluid, or other secretions) that can be sampled through minimally invasive or non-invasive methods (121). Blood-based liquid biopsy may include analysis of circulating nucleic acids (mainly cfDNA, which includes ctDNA in patients with cancer); circulating tumor cells (CTCs); and proteins (121). The ability to detect cancer-related analytes from blood has unique advantages, especially in cancer (or suspected cancer) cases where obtaining a tissue sample for traditional histological analysis might be particularly risky or difficult.

### Tumor Tissue Sampling and Analysis as the Current Standard of Care

The conventional path to achieving cancer diagnosis in companion animals varies based on patient characteristics, tumor type, and tumor location (122). Fine needle aspiration (FNA) cytology is less invasive and lower risk compared to biopsy, and FNA is often used to make a preliminary or definitive diagnosis, develop a treatment plan, and predict prognosis (123, 124). However, inconclusive results or misdiagnoses can occur with FNA due to low cellularity, artifact, necrosis, minimal exfoliation of certain cell types, lack of tissue architecture, etc. (124, 125). Also, not all tumors are easily accessible by FNA (such as deep-seated abdominal tumors, many intrathoracic tumors, and tumors of the central nervous system); and some tumors with high vascularization, or those which might seed the body wall (e.g., urinary tract), are not amenable for sampling by FNA.

If FNA cytology is attempted and is non-diagnostic or equivocal, more invasive methods (such as a traditional biopsy or exploratory surgery) are often employed to obtain tissue for analysis prior to making a definitive diagnosis and initiating treatment (126). Compared with FNA, biopsies and surgeries entail higher risks of morbidity and mortality, which are dependent upon the site of the suspected mass and the characteristics of the procedure. Such risks include infection, internal bleeding, fracture after bone biopsy, intestinal perforation with endoscopic biopsy, pancreatitis after pancreatic biopsy, collapse of vertebra at spinal surgery sites, non-diagnostic results, and in the worst cases, death (122, 127–137).

### Circulating Biomarkers

The clinical and cost challenges of tissue analysis have stimulated the search for “non-invasive” methods that rely upon analysis of biomarkers found in easily accessible body fluids, such as blood, urine, and secretions. Despite decades of research, few such methods have entered broad clinical use, with the exception of testing for hematological malignancies where blood-based cytology is part of the standard of care. Solid tumors, which make up most malignancies in both humans and dogs, have seen limited benefits to date from methods that employ circulating biomarkers, with cfDNA-based approaches currently showing the greatest promise for the future.

#### Protein Markers

In humans, blood-based testing has provided the opportunity to profile tumors to aid in the diagnosis of cancers, and to guide treatment decisions; and the earliest such tests have targeted protein markers (138, 139). A number of blood-based protein biomarkers have been used for human cancer screening and monitoring using immunohistochemical methods, including: CEA for colorectal cancer, PSA for prostate cancer, CA-125 for ovarian cancer, and alpha-fetoprotein (AFP) for hepatocellular carcinoma (140–143). Using similar ELISA (Enzyme-Linked ImmunoSorbent Assay) testing methods, recent attempts have been made to measure the concentrations of histone proteins that form the core of nucleosomes in order to detect the presence of cancer (144, 145). The nucleosome is the basic structural unit of DNA packaging, consisting of a segment of DNA wound around eight histone proteins. As cancer cells die, they release histone-bound DNA into circulation, whereupon the histone proteins can be separated and independently assayed (Figure 1).

However, biomarker assays based on circulating proteins suffer from high rates of false positives and false negatives, since the same proteins exist in circulation in healthy individuals and can be increased for reasons other than cancer, such as inflammation, sepsis, and trauma (146); also, these markers may not be significantly elevated in a significant proportion of individuals with even advanced stage cancer, reducing the potential for a highly sensitive test (146). Importantly in dogs, nucleosome concentration is also elevated in benign disease and in trauma (147–151), limiting its diagnostic utility for cancer (152). For these reasons, circulating protein markers have not been broadly adopted for cancer detection, and are more commonly used for monitoring cancer in cases where the level of the corresponding protein was shown to be already abnormal at the time of diagnosis.

In veterinary medicine, there has been interest in leveraging protein biomarkers such as thymidine kinase type 1 (TK1), canine C-reactive protein (cCRP), and alpha-fetoprotein receptor (RECAF) for canine cancer detection (153, 154); however, such protein biomarkers are not highly specific for canine cancer and can be elevated due to other reasons including immune-mediated hemolytic anemia, thrombocytopenia, and polyarthropathy (155). In human medicine, protein biomarkers such as RECAF and AFP have demonstrated limited sensitivity and specificity for cancer detection (143, 156).

#### Circulating Tumor Cells

CTCs are intact tumor cells originating in solid tumors that can sometimes be detected in circulation, a finding that has catalyzed a considerable body of research aimed at using CTCs for cancer detection. However, multiple studies in humans have demonstrated that even in metastatic disease, as many as 20% to over 50% of patients (depending on cancer type) have no detectable CTCs in the typical sample volume collected (157–162). Similar performance has been observed in metastatic canine cancer (163). As a result, CTCs have not seen broad clinical adoption, and remain primarily a research tool in both humans and dogs (164, 165).

#### Circulating Nucleic Acids

Over the past decade, circulating nucleic acids – in particular cfDNA – have emerged as the most promising class of circulating biomarkers for non-invasive detection and characterization of cancer. cfDNA, which includes ctDNA in cancer subjects, is the focus of the remainder of this review.

### cfDNA Origins and Characteristics

**Figure 6:**
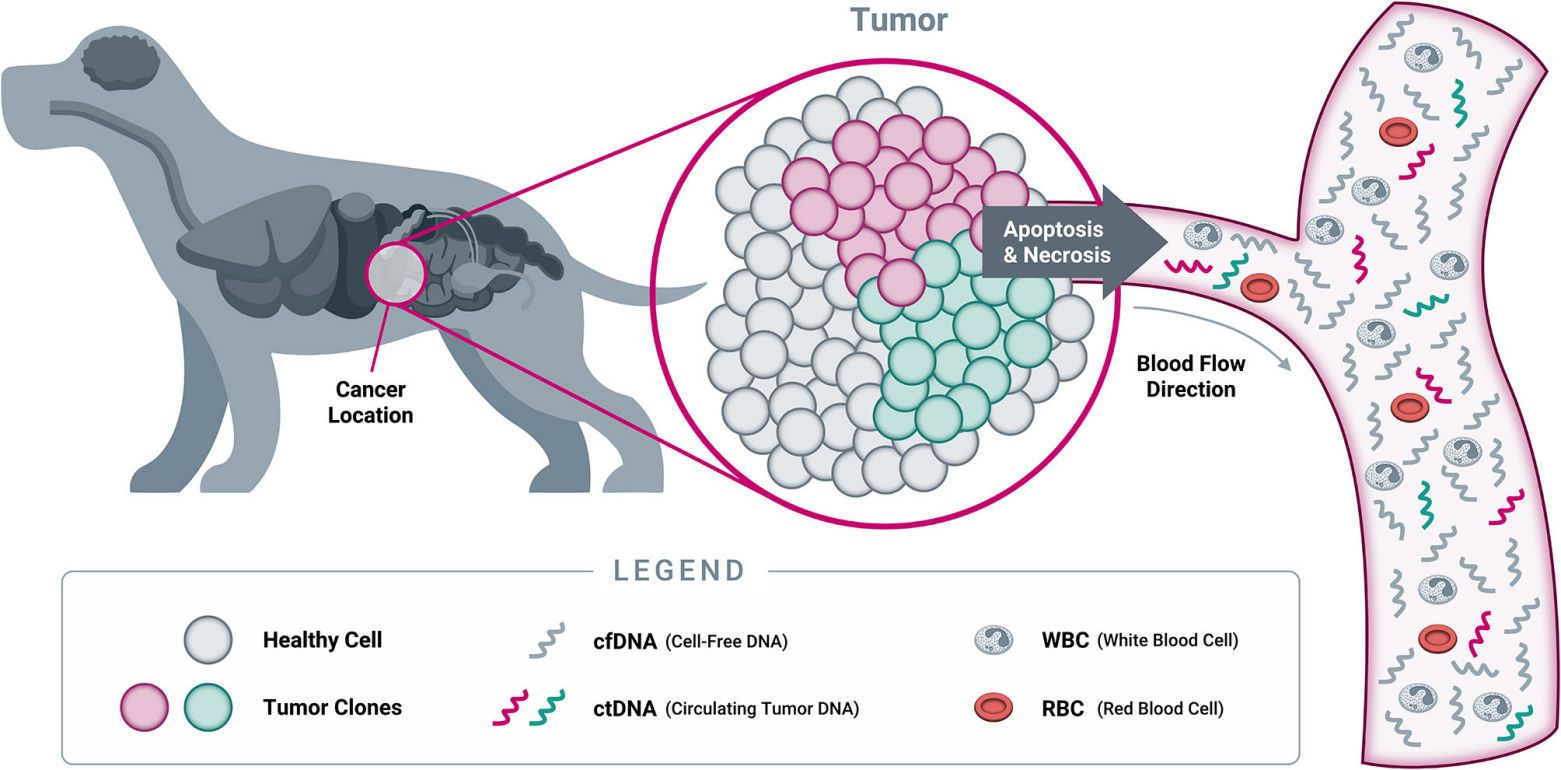
*Origins of cell-free DNA*. When a cell dies through either programmed cell death (apoptosis) or necrosis, its cellular contents (including DNA from the nucleus) are released into the bloodstream. At this point, the DNA becomes “cell-free DNA” and is rapidly degraded into small fragments through the action of circulating enzymes known as “DNAses.” As a result, most cfDNA fragments found in circulation are typically short, averaging 167 nucleotides in length in both humans and dogs (166, 167). While both healthy cells and tumor cells contain DNA that becomes cfDNA in circulation, only tumor cells will harbor somatic genomic alterations in cancer-related genes. Detection of such genomic alterations in the cfDNA of a patient is thus indicative of the presence of tumor cells in the body, providing the rationale for “liquid biopsy” testing approaches (Note: cfDNA exists as both single stranded DNA and double stranded DNA; only single stranded DNA is depicted here, for illustrative purposes).

As cells undergo programmed cell death (apoptosis) and necrosis, the membranes of cells and nuclei are broken down, and their contents are released into the circulation (Figure 6). Among these contents are fragments of DNA, known as “cell-free DNA” once they have left the confines of the cell and its nucleus. These cfDNA fragments are rapidly degraded by normal metabolic processes and have a very short half-life, estimated at 15 min to a few hours in both humans and dogs; as a result, they are usually cleared within a few days (168–170). The constant turnover of cells throughout the body provides a steady supply of cfDNA in the circulation, which is amenable to analysis with sophisticated technologies including NGS.

The presence of cfDNA in humans was first reported in 1948, and while cfDNA was hypothesized to be linked to metastatic cancer in the mid-1960s, it took until 1977 for the first results evaluating cfDNA concentrations in patients with cancer compared to normal controls to be published, and neoplastic characteristics were reported in circulation in 1989 (171–174).

In 1996, two landmark publications reported the detection of cancer-derived alterations in plasma or serum of cancer patients as ctDNA (175, 176). Since then, significant efforts have been devoted to developing molecular tests to detect the presence of cancer-derived alterations in the blood (161, 177), and use the information for cancer detection, characterization, treatment, and monitoring (27, 28, 101, 178–183).

In parallel, fetal-derived cfDNA was discovered in maternal plasma in 1997 (184), leading to the first widely adopted clinical application for cfDNA testing: a screen for common fetal chromosomal abnormalities such as trisomy 21 (Down syndrome) using a sample of the pregnant woman's blood (185). Prior to this revolutionary advance, such fetal genetic information could only be derived from invasive diagnostic tests such as chorionic villus sampling (CVS) or amniocentesis, which carry a risk of miscarriage (186). As a result, the introduction of cfDNA-based non-invasive prenatal testing (NIPT) in 2011 (185) fundamentally changed the way prenatal care is delivered. Tens of millions of pregnancies have been screened with this cfDNA-based technology to date, leading to a marked decrease in the number of invasive diagnostic procedures for detection of fetal chromosomal abnormalities (187).

There are many documented instances of NIPT results incidentally identifying maternal cancer, highlighting plasma as a common repository for both fetal-derived and cancer-derived cfDNA fragments (188, 189), and suggesting the potential of using plasma cfDNA to screen for asymptomatic cancers. Indeed, a population-based study published in 2017 reported the performance of cfDNA-based liquid biopsy to detect nasopharyngeal cancer before symptoms develop (190), which marked the first demonstration of using a cfDNA-based blood test to screen for a specific type of cancer. Multiple commercial providers are currently offering or developing liquid biopsy tests for human cancer applications, and many clinical trials are underway to expand the clinical utility of this technology to additional use cases and/or cancer types.

Published research on canine cfDNA has covered a variety of clinical applications, including trauma, sepsis, thromboembolism, and neoplasia, and has focused primarily on determining the concentration of cfDNA in plasma as correlated to a particular clinical state or as a predictor for certain clinical outcomes (84, 148–151, 167, 169, 191–203). Studies that evaluated cfDNA concentrations in healthy canine subjects have reported median concentrations ranging from less than 1 ng/mL to greater than 500 ng/mL (148, 149, 167, 169, 194–203)—significantly wider than the range documented in healthy humans (typically 0–20 ng/mL) (204). These wide-ranging findings suggest that additional research employing well-controlled, large-scale studies is required to better understand the fundamental characteristics of cfDNA in dogs; they also point to the need for standardized, reproducible methods for blood collection, extraction, and measurement of canine cfDNA. Such standardization will be critical for the successful transfer of cfDNA-based technologies such as liquid biopsy—currently limited to the human space where such methods are well established—to routine clinical use in veterinary medicine.

To provide the highest clinical value, a liquid biopsy test should be able to detect multiple classes of cancer-associated genomic alterations (described above) in cfDNA with high accuracy, even at very low concentrations in the circulation. Furthermore, the biology of cfDNA uniquely facilitates the evaluation of certain genomic features in circulation that can provide additional information about the presence and the origin of cancer.

For example, it is well-known that the attachment of methyl (CH3) groups to the DNA strand at specific locations throughout the genome is associated with cancer; methylation of the promoter regions of tumor suppressor genes can inactivate the expression of these genes, allowing oncogene-driven cancers to proliferate unopposed (205). Furthermore, DNA in cells from specific organs have methylation profiles that are specific to that organ (206). When DNA from cancer cells in a particular organ is released into circulation as ctDNA, its methylation “signature” carries information about the presence of cancer and about the organ of origin of that cancer (27, 207). For this reason, NGS-based analysis of cfDNA methylation profiles has emerged as one of the most promising approaches for detecting cancer and assigning it to a specific organ of origin, which has obvious clinical benefits (27).

Another unique feature of cfDNA is the fact that it is highly fragmented according to specific patterns. In the nucleus of a cell, DNA is organized in chromosomes as an uninterrupted strand ranging in size from tens of millions to over 100 million nucleotides (or bases). However, by the time it enters circulation following cell death and nuclear DNA degradation, cfDNA has been biologically degraded into fragments that are typically less than 1,000 nucleotides in length. In both humans and dogs, much of the cfDNA exists in fragments that are ~167 bases in length, representing the length of the DNA strand between two nucleosomes plus one full wrap of DNA around the histone proteins that make up the core of the nucleosome (166, 167, 208). Furthermore, it has been shown that in humans with cancer, the fragment length of cfDNA tends to be shorter; one of the key observations that have led to fragment profile analysis becoming an emerging method to improve the sensitivity for cancer detection. In addition, fragmentation features in cfDNA can also encode information about the organ of origin (209–213). As a result, fragmentomics – like methylomics – has the potential to extract unique information from cfDNA that points to both the presence of cancer and its organ of origin (214).

Emerging methylomic and fragmentomic methods leverage features that are unique to circulating tumor DNA and offer additional possibilities for the detection and characterization of cancer in circulation. However, the canine methylome has not been comprehensively characterized, which means that significant research will have to be performed before methylomics-based liquid biopsy solutions can be offered for oncology applications in dogs. Likewise, the canine cfDNA fragmentome is poorly understood at this time, requiring a massive investment in research to fully understand its potential for clinical use.

Currently, the only technology that can simultaneously interrogate all the major classes of genomic alterations in cfDNA, as well as features such as methylation and fragmentation patterns, is next generation sequencing (NGS). Leading liquid biopsy assays currently in use or under development in human medicine use advanced NGS-based techniques to evaluate a broad range of alterations and features across the genome that are known to be associated with cancer. Most of these approaches do not target a particular cancer type; instead, they take a “pan-cancer” approach rooted in the premise that cancer is fundamentally a disease of the genome, and accurate analytical detection of somatic genomic alterations will lead to accurate clinical detection of a wide variety of cancer types. Assays that combine multiple classes of genomic alterations and/or orthogonal genomic features are likely to yield improved clinical performance (such as higher sensitivity and specificity) or provide additional useful information (such as organ of origin prediction and identification of molecular targets for personalized treatment) across a broad range of cancer types. The past few years have also seen the debut of multi-omic liquid biopsy approaches that combine (for example) genomic and proteomic methods, breathing new life into protein analysis as a valuable adjunct to cfDNA analysis (26, 28). Similar combinatorial strategies will likely be required for the successful development of a pan-cancer liquid biopsy test for dogs.

### Clinical Use Cases and Clinical Utility of Liquid Biopsy in Cancer

**Figure 7:**
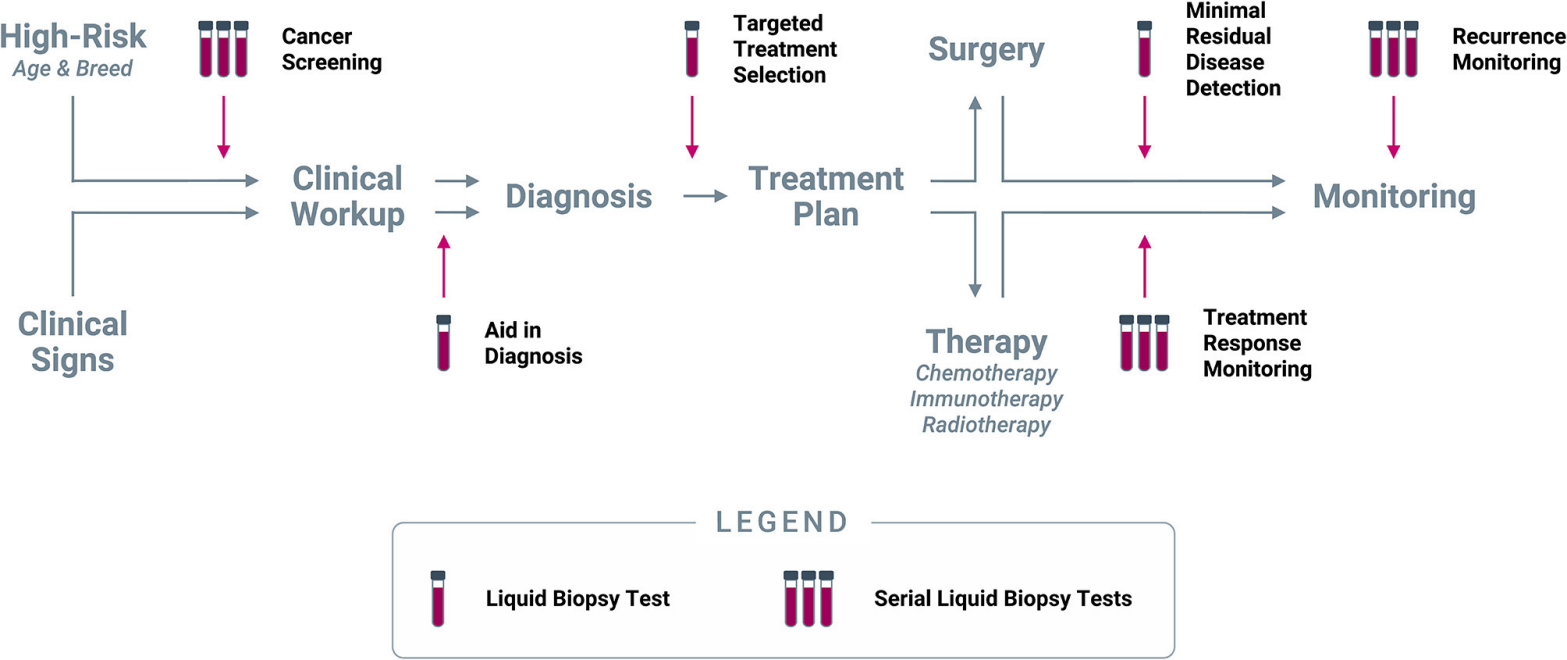
*Clinical use cases for liquid biopsy in cancer*. Liquid biopsy can be used to inform multiple decision points along the entire continuum of cancer care: (1) *Cancer screening* at regular intervals in patients deemed to be at higher risk for cancer based on age and/or breed; (2) *Aid in diagnosis* in patients who present with clinical signs (including incidental findings on imaging or laboratory tests) that are suspicious for cancer; (3) *Targeted treatment selection* based on the unique mutational profile of the tumor in patients diagnosed with cancer; (4) *Minimal residual disease detection* following a curative-intent intervention (such as surgery); (5) *Treatment response monitoring* at regular intervals during extended-duration therapeutic regimens; (6) *Recurrence monitoring* at regular intervals after complete remission or presumed cure.

Liquid biopsy promises the convenience of a blood draw combined with the power of genomic technology. It is unlikely to fully replace the key role that traditional tissue biopsy plays in veterinary cancer diagnosis and management, but the non-invasive nature of liquid biopsy, coupled with its ability to detect tumor signal from any malignant mass in the body, should allow it to provide immediate value in several clinical scenarios once it becomes commercially available. In humans, liquid biopsy has demonstrated feasibility and great clinical potential across multiple use cases, spanning the entire continuum of cancer care; a similar spectrum of applications is in principle available for veterinary uses of the technology (Figure 7).

Prior to a cancer diagnosis, liquid biopsy can provide valuable information in (1) presumably cancer-free patients as a *screening* test, and (2) in patients with clinical signs suspicious for cancer as an *aid in diagnosis*. Upon confirmation of a cancer diagnosis, liquid biopsy can be used to (3) identify a personalized treatment path based on the mutational profile of the tumor for *targeted treatment selection*; and (4) if the patient is to undergo a curative-intent intervention (such as a surgical procedure), a liquid biopsy immediately following the intervention can be used to test for *minimal residual disease*. After initiation of a longer-term therapy, such as chemotherapy or radiotherapy, liquid biopsy can be used (5) at regular intervals for *treatment response monitoring*. Finally, once a patient completes their course of treatment and is determined to be cured or in complete remission, liquid biopsy testing at longer intervals can be used for (6) *recurrence monitoring*. Each of these use cases, and their potential applicability in dogs, are described in more detail below.

#### Screening

Certain dog breeds are known to be more predisposed to cancer than others, presumably due to cancer-predisposing mutations that have become concentrated in the population over time as a result of the breeding process; however, the germline mutations responsible for most of these cancer predispositions are not as well-understood as in humans. It is also well-established that, just as in humans, cancer incidence in dogs increases with age (3). In a large fraction of cases, cancers in dogs are diagnosed at advanced stages after they have spread beyond the organ of origin, when prognosis is poor and the ability to extend life by treatment is limited (12–16). A liquid biopsy-based screening paradigm focused on high-risk populations, such as dogs from predisposed breeds or from geriatric populations, could help identify many of these cancers earlier. Early detection has been shown to drive better clinical outcomes in humans, such as increased life expectancy and higher rates of achieving complete remission following curative-intent interventions (e.g., surgery); historically, this has provided the rationale for well-established screening programs such as colonoscopy, mammograms, Pap smears, PSA screening, and low-dose CT scans (183, 215–217). Liquid biopsy solutions for universal cancer screening in humans are nearing commercialization (216, 218–220), and some of these assays have also shown potential for predicting the organ of origin of the tumor, facilitating the path to a definitive diagnosis (27, 28, 221–223).

State-of-the-art liquid biopsy assays currently in development for pan-cancer screening in humans have demonstrated detection rates (sensitivity) for early-stage cancer ranging from ~20 to 70% across multiple cancer types, at specificities of 98 to >99% (false positive rates of 2 to <1%) (27, 28, 211). High specificity is particularly important in cancer screening, given potential harms resulting from the diagnostic work-up of false positive screens, and from diagnosis and treatment of cancers that may never have become clinically apparent without screening (overdiagnosed and overtreated cases) (224). Screening results implying the possibility of a cancer diagnosis can also impose a considerable psychological burden on people who receive false positive results (225, 226), and it is reasonable to assume that pet owners would likewise experience distress as a result of false-positive cancer screening results in their companion animal.

A recent health economic modeling study revealed that adding an annual universal cancer screening test to the current standard of care in human medicine would reduce late-stage cancer incidence by 78% in those intercepted by the screening test, and result in an absolute reduction of 26% in all cancer deaths (227). The practice of screening at regular intervals relies on the concept of “cumulative detection” to improve the clinical sensitivity over time at the population level, as sequential testing holds the benefit of detecting cases missed on initial screening (108, 109, 228). Ultimately, this technology may support cancer screening in lower-risk canine populations as well, comparable to how NIPT technology expanded beyond high-risk cases to encompass all pregnancies in humans (229).

#### Aid in Diagnosis

One of the most common scenarios in which liquid biopsy may add value in the veterinary clinic is as an aid in diagnosis, when cancer is suspected due to clinical signs (including incidental findings on imaging or laboratory tests) or clinical history. Due to the high-risk nature of this patient population, this scenario is likely to provide the initial opportunity for liquid biopsy to be deployed in veterinary medicine. In some cases, clinical signs may be non-specific and not localizing to a certain anatomic site; whereas in other cases an anatomic site may be evident, but the invasive procedures required to obtain tissue for diagnosis may carry a high risk of complications, or the suspected mass is inaccessible by biopsy or surgery. In such cases, liquid biopsy could significantly shorten the time to a definitive diagnosis and help avoid the challenges typically associated with a long diagnostic odyssey. Often, elucidation of such clinical cases requires additional appointments, time, and expense; and diagnosis may be delayed or missed completely. Many pet owners may decline biopsy or exploratory surgery due to the associated risks and cost, missing the opportunity to obtain an adequate diagnosis and select an appropriate treatment. A liquid biopsy can be conveniently performed from a routine blood collection drawn during the initial visit when cancer is first suspected, potentially saving time and money while increasing compliance.

In both the screening and the aid in diagnosis use cases, liquid biopsy can facilitate earlier detection of cancer compared to the current standards of care. In addition to improving outcomes, earlier diagnosis can mitigate the financial burden of treatment, making it a cost-effective paradigm both at the population level and at the level of individual patients. Health economic studies have shown that treatment costs for human cancer patients diagnosed early in the disease course to be 2 to 4 times less than for those diagnosed at later stages (216, 217). Treatment for early-stage cancer typically consists of localized resection, which is often curative and has a short recovery time (28); whereas treatment for late-stage disease involves repeat courses of chemotherapy or radiation therapy aimed at extending life rather than achieving a cure. Availability of an affordable and convenient liquid biopsy testing option for proactive serial screening of dogs at high risk of cancer, or for first-line evaluation of canine patients suspected of cancer, could reshape the clinical and economic landscapes of pre-diagnostic cancer management in veterinary medicine.

#### Targeted Treatment Selection

In situations where surgical interventions are not feasible, other therapeutic options may be utilized, either with curative intent or as a chronic treatment to extend life and/or improve quality of life. In such cases, selection of a specific therapy may be based on established clinical practice guidelines; however, an emerging area in human medicine, often designated by the terms “precision medicine” or “personalized medicine,” aims to utilize the genomic signature of an individual's cancer to select specific targeted therapies (230, 231). For humans, there are over 200 FDA-approved drugs for the treatment of cancer (232) including a subset of more than 50 drugs matched (or “targeted”) to specific genomic alterations in a tumor, with many additional targeted-treatment candidates in various phases of development (233–235). For dogs, there are only two drugs that are FDA approved at the time of this writing for the treatment of cancer - toceranib (Palladia™) and tigilanol tiglate (Stelfonta®), with two more drugs - rabacfosadine (Tanovea®-CA1) and verdinexor (Laverdia™-CA1) - available under a conditional FDA approval (236). In the EU, the European Medicines Agency (EMA) has approved toceranib, tigilanol tiglate and mastinib mesylate (Masivet®) (237). Of these approved or conditionally approved drugs, only toceranib (a multi-kinase inhibitor that inhibits c-kit, PDGFR, and VEGFR2), and mastinib (a c-kit inhibitor) can be used as a targeted drugs linked to specific genomic features of a tumor, as improvements in tumor response (43, 238) and outcome (239) have been demonstrated for tumors with an activating kit mutation; however, many targeted drugs used to treat human disease are currently used off-label in dogs (236, 240). Many compounds developed (and FDA-approved) for use in humans underwent preclinical safety testing in dogs; significant safety and dosing data are thus available to help inform the treatment of canine cancer patients with these agents (241).

State of the art liquid biopsy approaches have the potential to comprehensively evaluate the genomic signature of a patient's cancer directly from blood – the final common pathway for ctDNA derived from all tumor subclones in the patient's body; this unique capability makes therapy selection based on liquid biopsy results less susceptible to treatment selection bias resulting from tumor heterogeneity, a bias that is unavoidable when a tumor is only sampled by a single tissue biopsy. Liquid biopsy results could be used for targeted treatment selection, especially for treatments where the genomic alteration targeted in humans has a direct ortholog in the canine genome. This could lead to more rapid and widespread utilization in canine cancer patients of targeted therapies currently approved for human use. The availability of liquid biopsy assays, as subject selection tools during the drug development process and as companion diagnostics following regulatory approval, can also accelerate the development of canine-specific targeted therapeutics; ultimately this will likely be the preferred path to bringing targeted treatments into veterinary oncology, as human-oriented targeted treatments might not have the same efficacy in canine cancer even if the targeted genomic alteration is perfectly homologous across the two species (242).

#### Minimal Residual Disease (MRD) Detection

After curative intent treatment (such as surgery) has been performed to remove the tumor, adjuvant therapy is often considered because of the risk of malignant deposits remaining in the body and resulting in relapse (or recurrence) in the future (243). MRD is defined as occult malignant disease that exists immediately after surgery and is undetectable by conventional methods; however, it can often be detected by the presence of ctDNA in the circulation (244, 245). The short half-life of cfDNA (minutes to hours in both humans and dogs) makes it an ideal analyte for MRD testing, as detection of any amount of ctDNA starting within a few days after surgery would point to the persistent presence of malignant disease in the body (168, 169). Many cancer types in humans have been studied in the context of MRD detection, including breast, pancreatic, lung, nasopharyngeal, and colorectal, as well as hematological malignancies (246, 247). In colorectal cancer for example, MRD detection has strong prognostic value, as patients with undetectable ctDNA post-operatively have significantly improved recurrence-free survival compared to those with detectable ctDNA in plasma (178, 244). In fact, detectable ctDNA post-operatively has a stronger prognostic association than many of the other traditional high-risk pathological and clinical features typically used by oncologists when considering adjuvant chemotherapy for patients with stage II colon cancer (246, 248). Similarly, the adoption of liquid biopsy-based MRD testing for canine patients could be used to inform the clinician about the relative risk of recurrence following curative-intent interventions, and thereby guide decisions regarding initiation of adjuvant treatment as soon as the patient has recovered from surgery.

#### Treatment Response Monitoring

Traditionally, treatment response monitoring has been performed by clinical observation and by imaging (mainly ultrasound and radiography, in the veterinary setting). Formalized procedures for documenting treatment response in dogs, such as the Canine Response Evaluation Criteria for Solid Tumors (cRECIST v1.0), have been published based on these methods (249, 250). However, reliance upon imaging alone for ascertaining treatment response has significant shortcomings. There are well-documented high inter-observer variabilities with imaging approaches in both dogs and humans, which can complicate the interpretation of imaging studies read by different radiologists (251–256).

In addition, hyperprogression (faster-than-expected tumor growth while under treatment) (257) and pseudoprogression (an initial apparent increase in tumor size or appearance of a new lesion on imaging during treatment, followed by tumor regression) (258, 259) can confound the interpretation of imaging for evaluation of treatment response. Lesion growth observed on imaging after treatment initiation may be due to advancing disease (secondary to ineffective treatment), an inflammatory response (resulting from tumor destruction by the treatment or from a direct side effect of the treatment), or simply ongoing tumor growth in the setting of a delayed treatment effect (257). Due to these complexities, real-time monitoring of tumor dynamics via serial liquid biopsy testing may help the clinician differentiate among these challenging scenarios and obtain more frequent updates on the patient's response to treatment than might be feasible with imaging alone.

The concentration of ctDNA in plasma can serve as a surrogate for the overall tumor burden (161), and patients with undetectable ctDNA after treatment are more likely to have had a complete response (178, 248, 260). Furthermore, the precise genomic variants in an individual's cancer can be used to follow the efficacy of the treatment in real time. This monitoring for treatment response may be useful regardless of the treatment modality (e.g., IV vs. oral chemotherapy, radiation, etc.). Since many chemotherapeutics are costly and typically require multiple clinic visits (20, 261), a ctDNA-based treatment response monitoring approach can offer significant value by detecting treatment response or treatment failure sooner than imaging or clinical observation would. This earlier detection may allow for early discontinuation of non-efficacious therapies in favor of alternate therapies that might have a better efficacy profile; or it may reassure the pet owner to continue a course of successful treatment even if clinical improvement is not readily apparent, or when a mixed clinical picture raises the question of disease progression vs. side effects of an otherwise efficacious treatment.

Monitoring for treatment response will also likely yield insights into the genomic evolution of tumor clones under the selective pressures of treatment—for example, the emergence of resistance mutations, or the emergence of new genomic variants potentially targetable by a different drug (262–264). Such molecular insights into tumor evolution are currently possible with standard tumor biopsy; however, even if molecular profiling of tumor tissue were widely available, longitudinal monitoring through repeat tissue biopsies would not be feasible in actual practice due to clinical, ethical, and financial considerations. Compared to current methods for monitoring treatment response, liquid biopsy would represent a complementary tool to better understand the evolution of the tumor, and its non-invasive nature could pave the way for liquid biopsy to become a routine monitoring test during cancer treatment in dogs.

#### Recurrence Monitoring

Even in patients who are thought to have achieved complete remission or a cure following successful treatment, the possibility of disease recurrence remains an ever-present concern. Sequential cfDNA testing during the post-treatment period aims to detect residual disease at a pre-clinical stage and flag a “molecular relapse” well before clinical relapse becomes otherwise evident (246). Many recent studies have described the use of liquid biopsy to identify human patients with molecular relapse many months before clinical or radiological relapse (246). Early identification of cancer relapse may help guide treatment and management decisions in canine patients as well, with the goal of improving clinical outcomes through earlier adjuvant therapeutic intervention.

## Discussion and a Look to the Future

Development of high-quality liquid biopsy tests for dogs comparable to those currently available for human testing has the potential to revolutionize the detection, characterization, and management of cancer in pets. However, the challenges involved in such development are significant. To observe cancer-related genomic variants at low concentrations in blood, the assay must interrogate a large number of cfDNA fragments, the majority of which will not be tumor-derived. This drives the need to focus on genomic regions of known clinical relevance for cancer. Pending results from large-scale discovery efforts across all major canine cancer types, these clinically-relevant genomic regions can only be identified from the—limited—available literature describing genomic alterations in canine cancers, or by homology mapping from the much more substantial human knowledge base. Identifying high-confidence orthologous regions in dogs for the top cancer-related regions in humans is non-trivial and will require significant effort and expertise.

After defining the genomic regions and features of interest, the process of developing a robust assay to detect low ctDNA signal presents a number of challenges, including: (1) optimizing best practices for the collection and isolation of cfDNA from canine plasma; (2) optimizing enrichment of targeted genomic regions; (3) maximizing the signal-to-background ratio of tumor-derived ctDNA vs. non-cancer cfDNA during data analysis; and (4) establishing a normal reference baseline, so that a signal indicative of cancer can be confidently segregated from random signals in patients without cancer who may have other clinical conditions that also could present with cancer-like signatures. For example, a well-documented challenge in the human liquid biopsy field is posed by the presence of clonal hematopoiesis of indeterminate potential (CHIP), also known as age related clonal hematopoiesis (ARCH) and defined as the accumulation of somatic mutations in hematopoietic stem cells that are clonally propagated to their progeny, a process that is associated with aging (265, 266). This phenomenon has not yet been documented in dogs, but it is reasonable to expect that it could also be a confounder in canine liquid biopsy, requiring sophisticated approaches to mitigate the impact on the false positive rate of such tests.

An analytically robust and clinically accurate liquid biopsy assay for use in canine patients will be highly complex, potentially generating billions of data points (base reads) for each test from NGS data; and will require extensive analytical and clinical validation to demonstrate reliability and clinical performance. Although the veterinary diagnostics space is not subject to the extensive regulations that apply to human diagnostics, it is imperative that any candidate liquid biopsy solution undergo validation at a level similar to that expected for human use, to maximize benefit for veterinary patients and clinicians. Clinical validation should be performed in adequately sized cohorts of canine subjects with a variety of cancers as well as presumably cancer-free canine subjects, to demonstrate both high sensitivity (few missed cases of cancer) and high specificity (few false positives). The results of such studies should be published in peer-reviewed journals so that the veterinary community is able to review the full corpus of supporting data before starting to use liquid biopsy tests in routine practice.

As liquid biopsy solutions become available in veterinary medicine, the clinical paradigm can be expected to shift in order to accommodate the inclusion of additional information afforded by the new modality; over time, veterinarians will develop an informed appreciation for the clinical utility of liquid biopsy in each care setting and incorporate this new tool judiciously into their clinical algorithms. Specifically, screening and aid in diagnosis will likely show the most immediate clinical utility for liquid biopsy by shifting diagnosis to an earlier timepoint when clinical outcomes are superior. In addition, the use of liquid biopsy for detection of minimal residual disease and for recurrence monitoring promises to provide an earlier opportunity to determine if a curative-intent intervention (i.e., surgery) was successful – and to inform the timely use of adjuvant treatments if the disease has not been eradicated. Finally, as more treatment options become available in veterinary medicine in the form of targeted therapies aimed at specific genomic alterations, the standard of care may evolve to include liquid biopsy as a routine pre-treatment selection step, and as a complement to imaging for evaluating response to treatment.

Liquid biopsy solutions based on cfDNA analysis are well-positioned to revolutionize certain aspects of cancer care in veterinary medicine by enabling safe, non-invasive testing at frequent intervals as dictated by the needs of each clinical case. However, liquid biopsy is not a panacea for all the challenges facing veterinary cancer management, and limitations exist. Certain tumors may not shed sufficient ctDNA into circulation to allow for confident detection and characterization of the disease by liquid biopsy; this can happen with smaller sized tumors in early disease, or with certain malignancies that tend to release lower levels of ctDNA into the bloodstream (such as tumors of the central nervous system) (161, 267). Also, the novelty of liquid biopsy means that extensive education will be required before its use can become widespread in the veterinary community, presenting a practical limitation to the speed and extent of adoption. Finally, the economics of a liquid biopsy-based approach to veterinary cancer diagnostics are yet unknown, which can present challenges – especially in the early years. In some use cases, such as aid in diagnosis when cancer is already suspected on clinical grounds, liquid biopsy may offer obvious cost advantages over invasive diagnostic procedures; in other cases, the economic value of liquid biopsy may be less apparent, such as with annual screening of dogs who will never go on to develop cancer, or with testing for targeted treatment selection when the only available options are off-label human therapeutics that have not been directly shown to be efficacious in canine cancer. Pricing considerations will certainly play an important role in the overall economics of the emerging liquid biopsy paradigm; ongoing decreases in the cost of sequencing, rapid improvements in assay design and automation, volume-driven economies of scale, and competition among providers should all contribute to favorable developments in pricing, making liquid biopsy an increasingly affordable testing option for pet owners.

Tumor tissue analysis is likely to remain a core component of the standard of care, especially for cases where malignant masses can be easily sampled by biopsy or surgery. Traditional tissue histopathology can provide unique and highly valuable information, such as: establishing a definitive diagnosis of cancer; determining aggressiveness and prognosis; and selecting a treatment – this being especially useful in cases where genomic analysis of the tumor does not provide any obvious targeted treatment options. As experience with liquid biopsy builds within the veterinary community, this new testing method may prove to be a replacement for older methods in some cases but is more likely to establish itself as a complementary or backup method alongside existing approaches, expanding the overall ability of the clinician to provide the most personalized care to each patient.

The genomic revolution has already had a marked impact on cancer care for human patients and is poised to revolutionize veterinary medicine in a similar manner in the coming years. As genomics becomes a routine part of veterinary care, expansion into *multi-omic* liquid biopsy approaches is likely to follow, including epigenomics (methylation and histone-modification analyses), transcriptomics (gene expression, micro RNAs), proteomics (tumor markers, other peptides), metabolomics, fragmentomics, etc. (121, 214). When combined, these orthogonal datasets will enable a multidimensional view of the cancer in real-time, enabling delivery of the highest quality of care. The introduction of high-quality, clinically validated pan-cancer liquid biopsy tests into the realm of veterinary medicine has the potential to substantially impact every step along the clinical journey of a canine cancer patient, from early detection to recurrence monitoring.

Long known as “man's best friends,” dogs live much shorter lives than humans, yet they form exceptionally close bonds with their human companions as well as with other pet dogs in the family; the loss of a pet dog often has a devastating emotional impact on the surviving family members, whether humans or other pet dogs (268–270). Cancer is by far the single most common cause of death in dogs, and having a pet companion that is fighting a losing battle with late-stage cancer is particularly difficult for families because of the financial strain of managing the disease in its final stages, and because the process is often drawn out over weeks or months and may involve considerable physical pain for the patient (271–273). The decision to euthanize a pet family member is one of the most difficult decisions a family will make. As veterinary medicine stands on the threshold of the new era of genomic medicine, novel tools – convenient, affordable, non-invasive, and widely available – will enable veterinarians to routinely screen for cancer and detect it early, when it can be cured; pursue rapid diagnosis of cancer as soon as the disease is suspected; and select targeted treatments and monitor for response and recurrence after a diagnosis has been made. These new tools will allow countless families to spend more time with their beloved pet family members and will further empower veterinarians to honor their professional oath to use their “scientific knowledge and skills for the benefit of society through the protection of animal health and welfare. [and] the prevention and relief of animal suffering” (274).

Humans have benefited extensively from medical advances that were first trialed in our canine sidekicks. By implementing lessons learned from recent genomic advances in cancer care for humans, we can now raise the level of cancer care for our canine companions as well. It is fitting to consider that widespread adoption of liquid biopsies in veterinary medicine may represent an upcoming historic opportunity to repay our “best friends” for their many prior contributions to our well-being.

## Author Contributions

JC and DG contributed to conception and design of the manuscript and co-wrote the first draft. All authors contributed to the manuscript and provided critical revisions, read, and approved the submitted version.

## Conflict of Interest

JC, AF, KK, IC, JT, KL, LH, DT, and DG are employed by or affiliated with PetDx. JC, AF, KK, NL, AN, ND, DB, TJ, JF, MS, IC, JT, KL, LH, MM, LD, DT, and DG hold vested or unvested equity in PetDx. TJ is employed by Laboratory Corporation of America. JF is Managing Partner at Friedman Bioventure, Inc. MS is Managing Director at RS Technology Ventures LLC. KK is an inventor on multiple patent applications related to bioinformatics methods for cancer diagnostics and holds equity in Illumina. MM is an inventor on multiple patent applications covering technologies for canine and human cancer diagnostics, and has licensing or consulting relationships with PetDx, Exact Sciences, AstraZeneca, Bristol Myers Squibb, and TGen. LD is a member of the board of directors of Personal Genome Diagnostics (PGDx) and Jounce Therapeutics. LD is a compensated consultant to PGDx, 4Paws (PetDx), Innovatus CP, Se'er, Kinnate and Neophore. LD is an uncompensated consultant for Merck but has received research support for clinical trials from Merck. LD is an inventor of multiple licensed patents related to technology for circulating tumor DNA analyses and mismatch repair deficiency for diagnosis and therapy from Johns Hopkins University. Some of these licenses and relationships are associated with equity or royalty payments directly to Johns Hopkins and LD. LD holds equity in PGDx, Jounce Therapeutics, Thrive Earlier Detection, Se'er, Kinnate and Neophore. LD's spouse holds equity in Amgen. The terms of all these arrangements for LD are being managed by Johns Hopkins and Memorial Sloan Kettering in accordance with their conflict of interest policies. The remaining author declares that the research was conducted in the absence of any commercial or financial relationships that could be construed as a potential conflict of interest.

## Abbreviations

cfDNA: Cell-Free DNA
CNV: Copy Number Variant
ctDNA: Circulating Tumor DNA
CTC: Circulating Tumor Cell
DNA: Deoxyribonucleic Acid
FNA: Fine Needle Aspiration
MRD: Minimal Residual Disease
NGS: Next Generation Sequencing
SNV: Single Nucleotide Variant
TDT: Tumor Doubling Time
TMB: Tumor Mutational Burden.
